# Supplementary figures and images for: Modulation of the E-cadherin in human cells infected in vitro with Coxiella burnetii
Source: PLoS One. 2023 Jun 7;18(6):e0285577. doi: 10.1371/journal.pone.0285577 (PMC10246793; doi:10.1371/journal.pone.0285577)

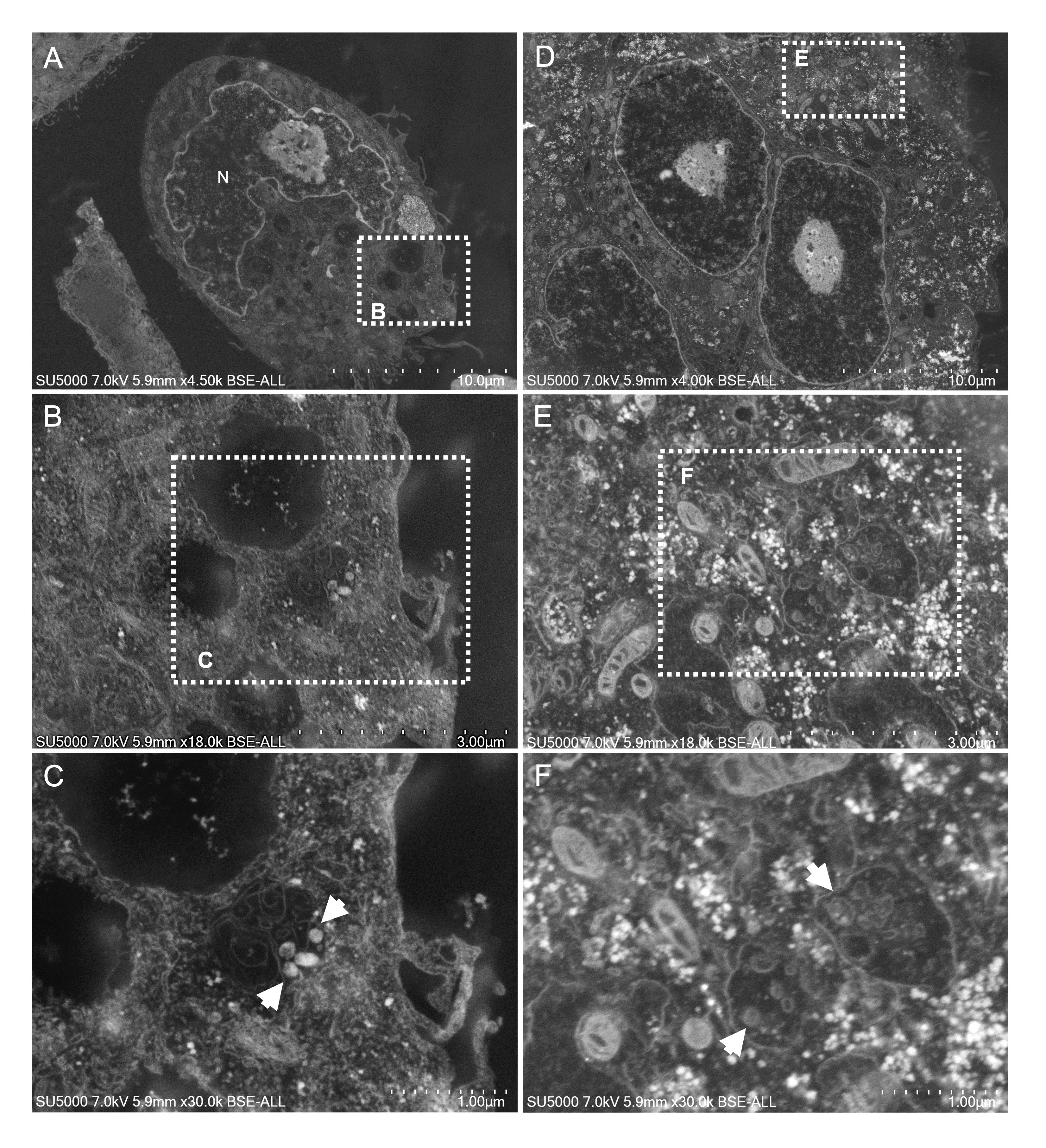

Supplement: S1 Fig — (A, B, C) SEM images of C. burnetii Guiana strain infected BeWo cells. (B) High magnification of the boxed region in (A) shows serval vacuoles dispersed in the cell cytoplasm. (C) High magnification of the boxed region in (B) shows a vacuole containing electron-dense circular structures resembling C. burnetii (white arrows). (D, E, F) SEM images of C. burnetii Nine Mile strain infected BeWo cells. (D) High magnification of the boxed region in (E) shows vacuoles inside the BeWo cell (F) filled with hypo electron-dense C. burnetii-like structures with or without less electron-dense internal content (white arrows). (JPG) [file pone.0285577.s001.jpg]

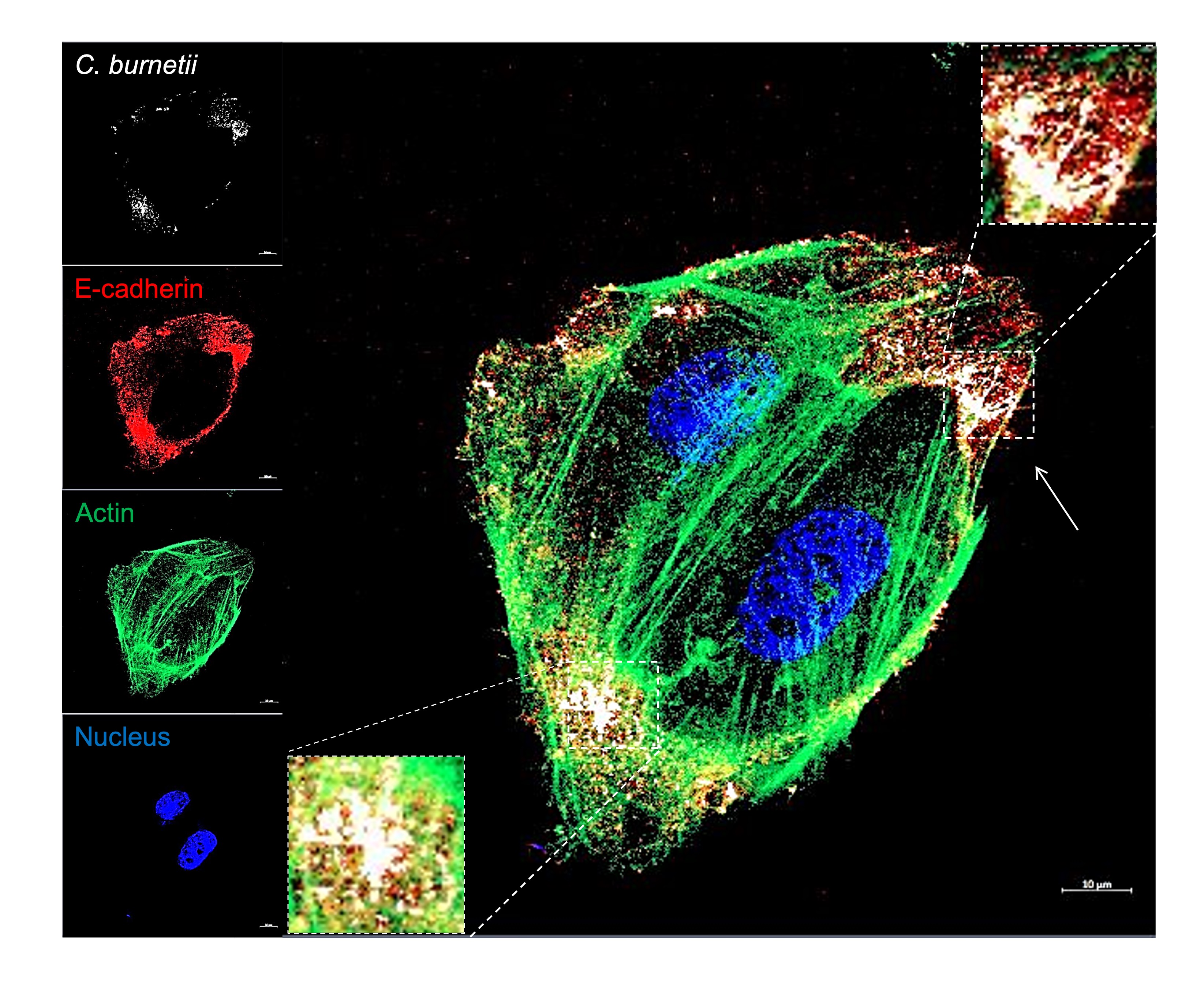

Supplement: S2 Fig — Fluorescence labelling was as follows: C. burnetii (white), E-cad (red), actin (green), and cell nucleus (blue). Images were acquired using a confocal microscope (Zeiss LSM 800) with a 63X/1.4 oil objective (scale bar: 10 μm). Regions of high concentration of C. burnetii are shown with higher magnitude within scales. (JPG) [file pone.0285577.s002.jpg]

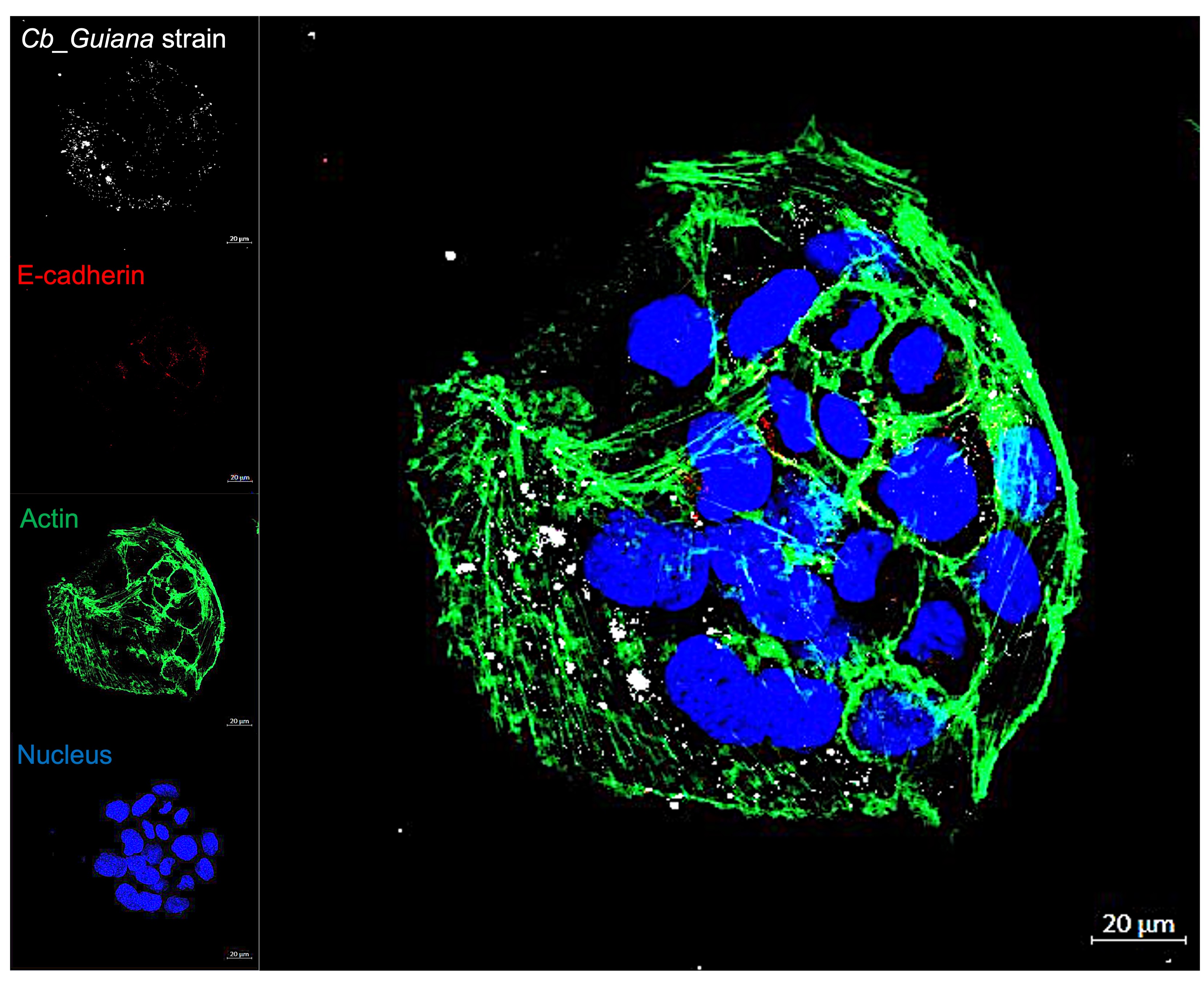

Supplement: S3 Fig — Fluorescence labelling was as follows: C. burnetii (white), E-cad (red), actin (green), and cell nucleus (blue). Images were acquired using a confocal microscope (Zeiss LSM 800) with a 63X/1.4 oil objective (scale bar: 20 μm). (JPG) [file pone.0285577.s003.jpg]

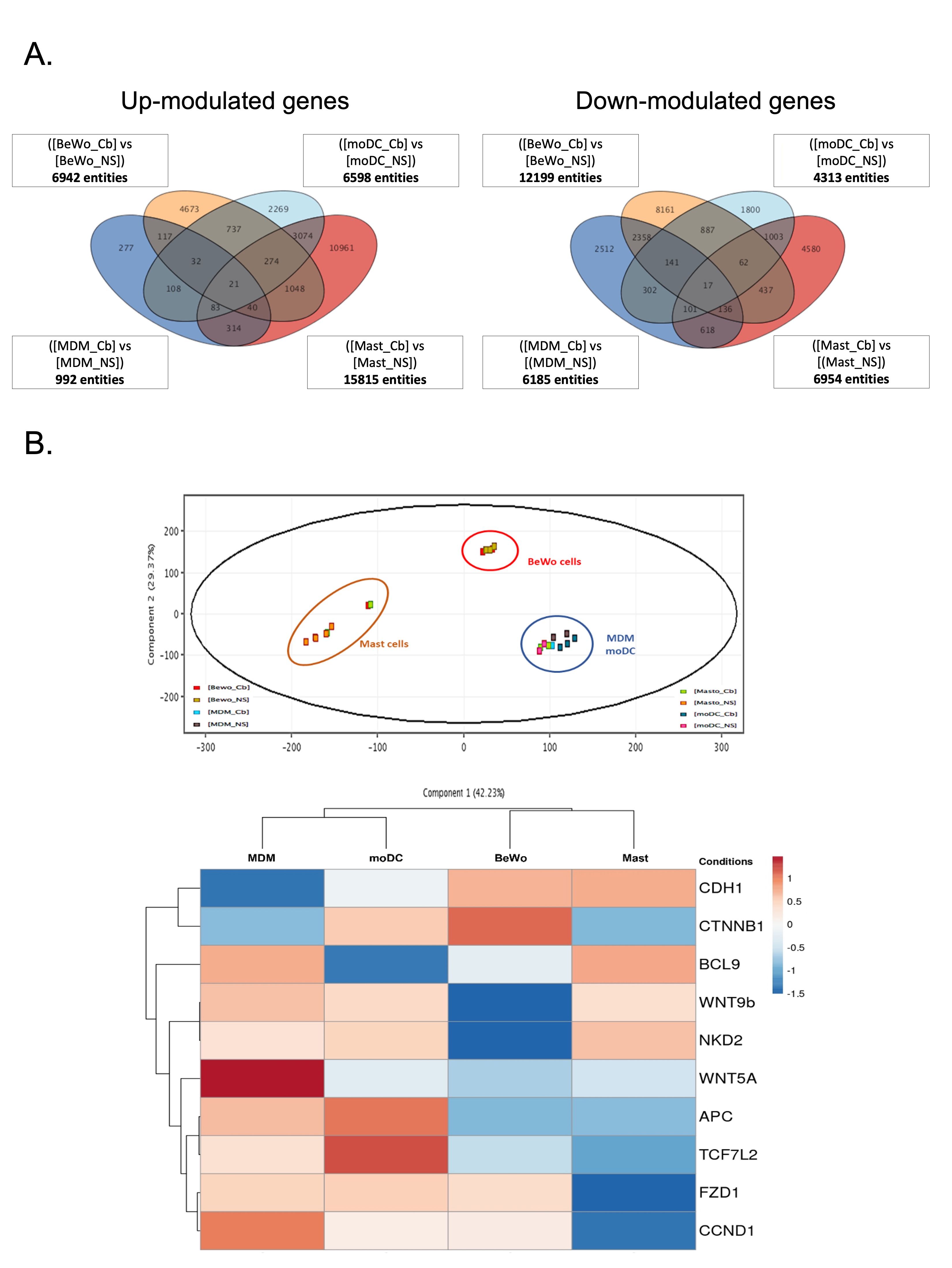

Supplement: S4 Fig — BeWo cells infected or not by C. burnetii were submitted to microarray analysis as well as primary cells, including monocyte-derived macrophages (MDM), monocyte-derived dendritic cells (moDC), and mast cells. (A) Set of genes up- or down-modulated in the panel of C. burnetii infected cells compared to uninfected cells (e.g., 6942 genes up-regulated and 12199 genes down-regulated in BeWo cells after C. burnetii infection). (B) principal component analysis and (C) hierarchical clustering were performed to show the distribution of the different groups of infected cells and the modulation of gene expression. (JPG) [file pone.0285577.s004.jpg]

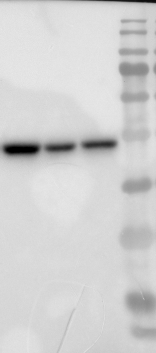

Supplement: S1 File — (TIF) [file pone.0285577.s005.tif]

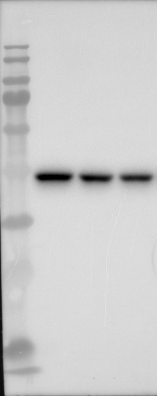

Supplement: S2 File — (TIF) [file pone.0285577.s006.tif]

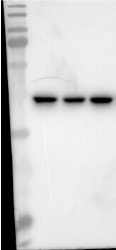

Supplement: S3 File — (JPG) [file pone.0285577.s007.jpg]

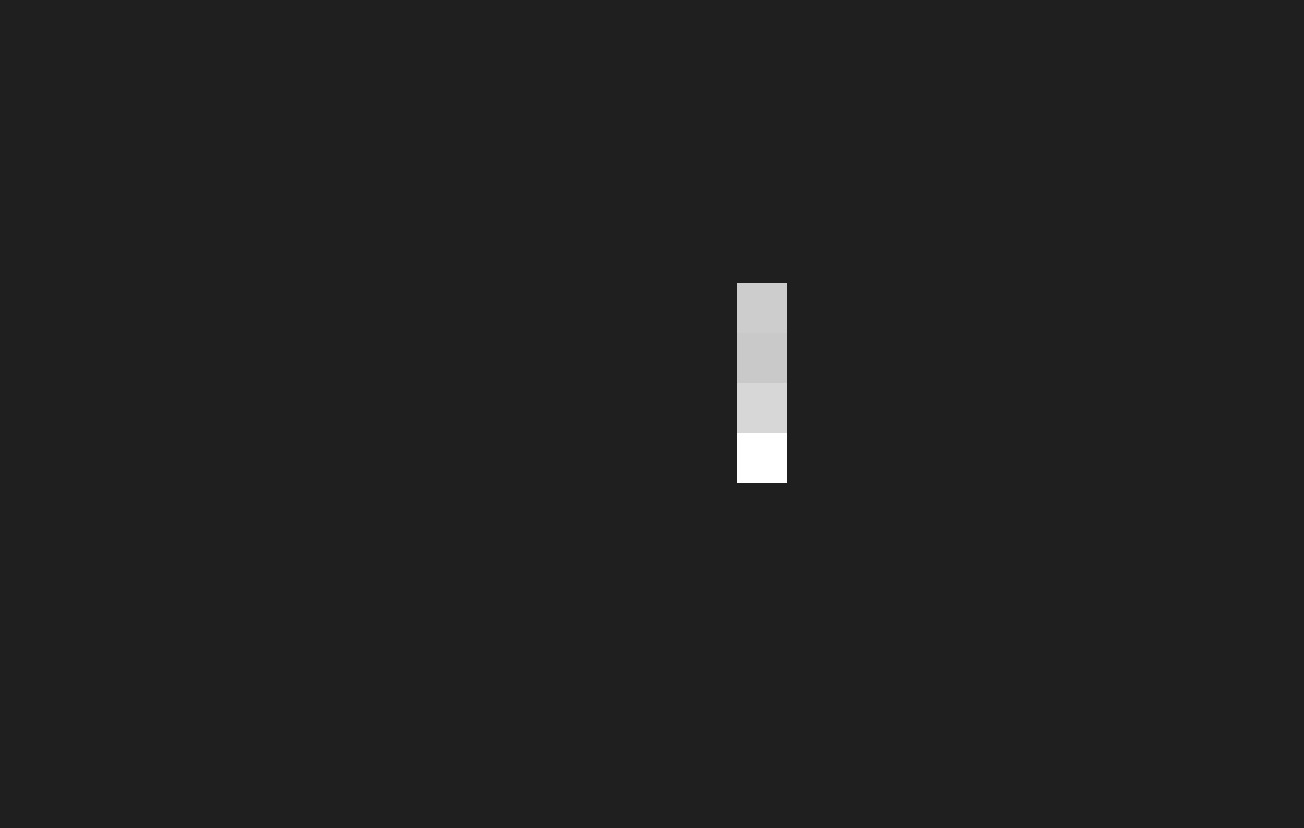

Supplement: S4 File — (JPG) [file pone.0285577.s008.jpg]

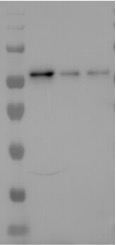

Supplement: S5 File — (JPG) [file pone.0285577.s009.jpg]

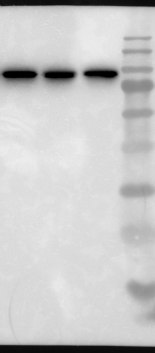

Supplement: S6 File — (TIF) [file pone.0285577.s010.tif]

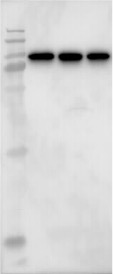

Supplement: S7 File — (JPG) [file pone.0285577.s011.jpg]

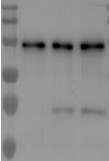

Supplement: S8 File — (JPG) [file pone.0285577.s012.jpg]

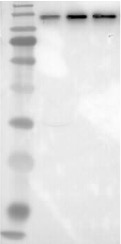

Supplement: S9 File — (JPG) [file pone.0285577.s013.jpg]

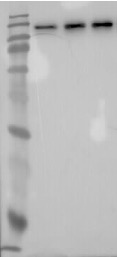

Supplement: S10 File — (JPG) [file pone.0285577.s014.jpg]
